# The Effect of Language Learning Strategies on Proficiency, Attitudes and School Achievement

**DOI:** 10.3389/fpsyg.2017.02358

**Published:** 2018-01-11

**Authors:** Anita Habók, Andrea Magyar

**Affiliations:** Institute of Education, University of Szeged, Szeged, Hungary

**Keywords:** language learning strategy, foreign language attitude, foreign language mark, general school achievement, lower secondary students

## Abstract

This study examines language learning strategy (LLS) use in connexion with foreign language attitude, proficiency and general school achievement among lower secondary students in Years 5 and 8 (*n* = 868) in Hungary. An adapted version of the Strategies Inventory for Language Learning questionnaire was used for data collection. The results showed that Hungarian students mainly engage in metacognitive strategies in both years. Differences between more and less proficient language learners’ strategy use have also been found. With regard to the effect of LLS on foreign language attitude, the foreign language mark and school achievement, path analysis indicated a good fit in both years. The metacognitive, social and memory strategies primarily influenced foreign language attitudes and marks in Year 5. The metacognitive strategies had a slight impact on school achievement as well as on foreign language marks. We demonstrated the dominant effect of metacognitive strategies and the low effect of memory strategies in Year 8. In addition, metacognitive strategies also influenced foreign language marks. The effect of foreign language marks on school achievement was also remarkable. There was a strong impact on the children’s attitudes through these variables.

## Introduction

In recent decades, a number of studies have focused on foreign language learning, with the emphasis often having been placed on language learning strategies (LLS; Wong and Nunan, 2011; Oxford, 2016). Several studies have confirmed that these strategies aid students in becoming more effective learners inside the classroom and foster more efficient development of students’ mastery of the target language after leaving school (Wong and Nunan, 2011). However, less is known about the structure and relationship between LLS, foreign language attitude, the foreign language mark and general school achievement (GA). Recent studies have mainly dealt with LLS among university students and upper secondary students, with only a few investigations having been conducted among lower secondary students. In the present study, we aim to examine young Hungarian students’ LLS use and its connexion to foreign language attitude, the foreign language mark and school achievement at the beginning and end of lower secondary school. We believe that it adds value to the article that we have investigated a young age group, as the beginning period of language learning can establish the success of the entire process. Another advantage of our research is that we analysed the whole language learning process in connexion with several other factors to represent the complexity of the language learning process.

## Theoretical Background

Studies on LLS in recent decades have identified a large number of strategies which are employed by English as a foreign/second language (EFL/ESL) learners and several strategy categorisation patterns have also been established. The most frequently used taxonomy was developed by Oxford (1990). She identified three direct and three indirect strategy types. Direct strategies are specific means of language use: memory, cognitive and compensatory (or compensation) strategies. Indirect strategies, such as metacognitive, affective and social strategies, support LLS indirectly. Recently, Oxford revisited her strategy categories and developed a model with four different strategy categories: cognitive, affective and sociocultural-interactive as well as a master category of “metastrategies.” Metastrategies comprise metacognitive, meta-affective and meta-sociocultural-interactive strategies (Griffith and Oxford, 2014; Oxford, 2016). However, she did not elaborate on this strategy classification, and thus our study relied on her original taxonomy.

Various studies have focused on LLS use and aimed to identify the strategies most frequently employed by language learners (Chamot, 2004; Magogwe and Oliver, 2007; Wu, 2008; Chen, 2009; Al-Qahtani, 2013; Charoento, 2016; Alhaysony, 2017; Dawadi, 2017). Overall, it can be concluded that the most commonly used LLS in these studies were metacognitive, compensation and cognitive strategies. However, Chamot (2004) pointed out that different strategy preferences were reported by students in different cultural contexts. Chinese and Singaporean students reported a higher level preference for social strategies and lower use of affective strategies than European students.

Some studies have dealt with the implementation of the SILL with a focus on school-aged students (Magogwe and Oliver, 2007; Chen, 2009, 2014; Gunning and Oxford, 2014; Platsidou and Kantaridou, 2014; Pfenninger and Singleton, 2017). The overall conclusion of these studies has been that young learners mostly used social, affective and compensation strategies. The use of memory strategies was relatively low (Doró and Habók, 2013). The attitudes of learners at this age toward language learning are particularly important since they can greatly determine motivation, learning outcomes and later success in language learning (Platsidou and Kantaridou, 2014; Platsidou and Sipitanou, 2014).

As the purpose of investigating LLS is to foster learning processes and improve language level, research projects often deal with LLS use in relation to language learning proficiency (Khaldieh, 2000; Magogwe and Oliver, 2007; Wu, 2008; Chen, 2009; Liu, 2010; Al-Qahtani, 2013; Platsidou and Kantaridou, 2014; Charoento, 2016; Rao, 2016). The notion of proficiency has been defined and involved in analysis in a multitude of ways by various researchers. Charoento (2016) involved self-ratings, Wu (2008) used the results from language proficiency and achievement tests, Magogwe and Oliver (2007) incorporated language course grades into their analysis of their results. Most studies have shown a positive relationship between LLS and proficiency, but the direction of their connexion was often different. Some researchers have stressed that strategy use was mainly specified by proficiency. More proficient students engaged in LLS more frequently and also employed a broader range of strategies overall compared to less proficient students (Khaldieh, 2000; Wu, 2008; Rao, 2016). Al-Qahtani (2013) and Charoento (2016) demonstrated that successful students mainly used cognitive strategies, while Wu (2008) emphasised significant utilisation of cognitive, metacognitive and social strategies among more proficient university students. Chen (2009) pointed to the use of fewer communication strategies among proficient learners, but noted that they employed them more efficiently than less proficient learners. In addition, Magogwe and Oliver (2007) also established that the basic difference in LLS use between proficient and less proficient learners was that more successful students not only used certain LLS significantly more often, but were also able to select the most adequate strategies depending on the goal of their task.

Some studies have dealt with the effect of LLS use on language proficiency. Both Liu (2010) and Platsidou and Kantaridou (2014) pointed out that learning strategy influences language use and that it plays a significant role in anticipating perceived language performance. Wu (2008) noted that cognitive strategies have the most dominant influence on proficiency. Rao (2016) found that students’ English proficiency significantly affected their learning strategy use and also observed that high-level students avail themselves of more strategies more frequently than low-level students.

Another essential area of LLS research is the study of strategy use in relation to affective variables, such as attitude and motivation (Shang, 2010; Jabbari and Golkar, 2014; Platsidou and Kantaridou, 2014). Most of these studies have found that learners with a positive attitude employed LLS more frequently compared to learners with a negative attitude. Platsidou and Kantaridou (2014) reported that attitudes toward second language learning influence both direct and indirect strategy uses and that changing learners’ attitudes toward language learning can thus foster their strategy practises. Jabbari and Golkar (2014) established that learners with a positive attitude employ cognitive, compensation, metacognitive and social strategies more frequently.

It can be concluded that LLS use has been studied extensively in recent decades. Most research has found that LLS cannot be analysed separately; it must be examined in relation to certain other factors, among which foreign language attitudes and proficiency play a central role (Griffiths and Incecay, 2016). However, most previous studies preferred university students or adults to primary or secondary school-aged students. Furthermore, a limited amount of research has investigated the relationship of LLS with attitude toward foreign language learning and the foreign language mark. There has also been a dearth of scholarship on how language proficiency and school achievement are determined by LLS use and attitude. Our study aims to fill this gap and attempts to present a comprehensive view of the relationship between LLS use and language attitude and between proficiency and general school achievement by focusing on school children at the beginning and end of lower secondary school. Our specific research question we focus on in this paper is the following:

What are the lower secondary school children’s strategy use preferences and how these are connected with their foreign language attitude, proficiency and general school achievement? Based on the relevant literature we assume that students of this age mainly employ indirect strategies, such as affective, metacognitive and social strategies and these have a significant impact on their foreign language learning attitude, proficiency and general school achievement.

## Materials and Methods

### Participants

The participants in the present study were lower secondary students (11- and 14-year-olds) in Hungary (*n*_Year5_ = 450, *n*_Year8_ = 418). Participation in the study was voluntary both for schools and students. This study was carried out in accordance with the recommendations of the University of Szeged, the Hungarian law and the municipalities that maintain the schools. The IRB of the Doctoral School (University of Szeged) specifically approved this research project. The agreements are documented and stored in written form in the schools.

Our target group generally started learning a foreign language in Year 4. As one portion of our sample have been learning a foreign language for at least four years, they must have experience of how they learn language. In Hungary, the primary level of education is composed of the elementary and lower secondary school levels; hence, the transition occurs with relatively few major changes, and children have the same language teacher during these school levels. While the foreign language teacher does not change, the other school subjects are taught by specialist teachers as of Year 5. Learning difficulties and differences among children grow considerably from the beginning of lower secondary school; hence, diagnosing language learning attitude is particularly essential.

### Instruments

The Strategy Inventory for Language Learning (SILL, Oxford, 1990) was administered to investigate the children’s LLS use. The SILL is a standardised measurement tool, and it is applicable to various foreign languages. The complex questionnaire is clustered into six strategy fields: (1) memory (9 items); (2) cognitive (14 items); (3) compensation (6 items); (4) metacognitive (9 items); (5) affective (6 items); and (6) social strategies (6 items). The participants were asked to respond to each statement on a five-point Likert scale. The answers ranged from ‘1 = never or almost never true of me’ to ‘5 = always or almost always true of me.’ The reported internal consistency reliabilities of the questionnaires ranged between 0.91 and 0.94 (Cronbach’s alpha) (Oxford and Burry-Stock, 1995; Ardasheva and Tretter, 2013). The questionnaire was conducted in Hungarian to eliminate differences in English knowledge and make it suitable for the language levels in these age groups. The reliability of the Hungarian version was confirmed in previous research (Doró and Habók, 2013). In addition, the children were asked to self-report their foreign language attitude, foreign language mark (indicating students’ foreign language knowledge) and general school achievement (grade point average, which includes students’ achievement in all subjects) on a five-point scale. In Hungarian schools, the different proficiency levels are rated on a five-point scale: 1 is the weakest mark, and 5 is the most excellent.

### Design and Procedure

Quantitative research design was employed through online survey methodology. The SILL questionnaire was administered via the eDia online testing platform, which was developed by the Centre for Research on Learning and Instruction for assessing Year 1–6 children’s foreign language knowledge and attitudes. One school lesson was provided for data collection; however, the children needed approximately 20 min to hand in their ratings. Both the children and teachers are familiar with this system because the online platform has been in use since 2009.

Data were handled confidentially during the testing procedure; the children used an identification code provided by research administrators. The researchers were only able to see the codes, and only the teachers were able to identify their students with the codes. All the instructions were in the online questionnaire, so the children were able to answer the questions individually. The teachers were also requested to report the children’s questions, remarks and difficulties during testing. Finally, the teachers reported no misunderstandings or problematic items during data collection.

The data analyses were twofold. First, SPSS for Microsoft Windows 20.0 was employed for classical test analysis, which included an estimation of frequencies, means and standard deviations. The significance of differences among the variables was determined by ANOVA analysis. Second, path analysis was managed by the SPSS AMOS v20 software package to analyse the effect of strategy use on the variables under observation (Arbuckle, 2008). The model fit was indicated by the Tucker–Lewis index (TLI), the normed fit index (NFI), the comparative fit index (CFI) and the root mean square error of approximation (RMSEA) (Byrne, 2010; Kline, 2015).

## Results

### Descriptive Analysis

#### General Strategy Uses among Lower Secondary School Children

The mean scores and standard deviations showed moderate LLS use, with the use of metacognitive, affective and social strategies being the highest in Year 5 (**Table 1**). Compensatory strategies were employed significantly the lowest. In Year 8, besides metacognitive and social strategies, cognitive strategies were relied on the most. Metacognitive strategy use was similarly high in both age groups. Significant differences were found between the age groups in memory, compensation and affective strategies (*p* ≤ 0.01). While the use of affective strategies was relatively high in Year 5, it was the least frequently employed in Year 8.

**Table 1:** The strategy use results for the sample.

	Year 5		Year 8		*F*	*p* <
Strategy	Mean	*SD*	Mean	*SD*		
Memory	2.96^∗^	0.88	2.68^∗^	0.73	12.284	0.001
Cognitive	2.90^∗∗^	0.88	2.79^∗∗^	0.83		n.s.
Compensation	2.72	0.93	2.69	0.78	12.649	0.001
Metacognitive	3.13	0.99	3.03	0.96		n.s.
Affective	3.02^∗^	1.04	2.63^∗^	0.87	10.444	0.01
Social	2.98	1.08	2.86	0.98	4.569	0.05

*^∗^p < 0.001, ^∗∗^p < 0.05.*

#### Differences in Strategy Use among Students with Different Proficiency Levels

One of our goals was to identify students’ LLS use preferences according to their proficiency levels. To implement this goal, we grouped the children into categories according to their proficiency, which was derived from their foreign language marks.

We combined the foreign language marks for those children who were evaluated with a 1 or a 2. These children showed a very low knowledge level and demonstrated a large number of difficulties and misunderstandings in foreign language learning. The next group was formed of children who were assessed at mark 3. This mark indicated an average knowledge level with gaps. Children who were evaluated with a mark 4 had fewer significant deficits. Children who received a mark 5 were the highest performers in school. **Tables 2**, **3** summarise our results on strategy use according to foreign language marks. The number of children is also indicated according to each category.

**Table 2:** Means of strategy users according to their foreign language mark in Year 5.

Strategy	Foreign language mark	Mean	*F*	*p* <	Sig.
Memory	1 or 2	2.42	13.061	0.001	{1; 2 < 3}
	3	2.88			{1; 2 < 4}
	4	2.96			{1; 2 < 5}
	5	3.21			{3 < 5}
Cognitive	1 or 2	2.51	9.385	0.001	{1; 2 < 4}
	3	2.75			{1; 2 < 5}
	4	2.90			{3 < 5}
	5	3.15			
Compensation	1 or 2	2.52	2.234	n.s.	
	3	2.69			
	4	2.66			
	5	2.86			
Metacognitive	1 or 2	2.68	15.607	0.001	{1; 2 < 4}
	3	2.83			{1; 2 < 5}
	4	3.08			{3 < 5}
	5	3.50			{4 < 5}
Affective	1 or 2	2.78	2.596	n.s.	
	3	2.89			
	4	3.03			
	5	3.17			
Social	1 or 2	2.58	11.704	0.001	{1; 2 < 5}
	3	2.73			{3 < 5}
	4	2.86			{4 < 5}
	5	3.35			

**Table 3:** Means of strategy users according to their foreign language mark in Year 8.

Strategy	Foreign language mark	Mean	*F*	*p* <	Sig.
Memory	1 or 2	2.50	7.708	0.001	{1; 2 < 5}
	3	2.48			{3 < 4}
	4	2.76			{3 < 5}
	5	2.87			
Cognitive	1 or 2	2.34	36.072	0.001	{1; 2 < 4}
	3	2.48			{1; 2 < 5}
	4	2.81			{3 < 4}
	5	3.31			{3 < 5}
Compensation	1 or 2	2.47	9.012	0.001	
	3	2.53			{1.2 < 5}
	4	2.71			{3 < 5}
	5	2.96			
Metacognitive	1 or 2	2.39	47.580	0.001	{1; 2 < 4}
	3	2.67			{1; 2 < 5}
	4	3.11			{3 < 4}
	5	3.68			{3 < 5}
					{4 < 5}
Affective	1 or 2	2.35	3.512	0.05	
	3	2.65			{1; 2 < 4}
	4	2.71			{1; 2 < 5}
	5	2.71			
Social	1 or 2	2.41	19.916	0.001	{1; 2 < 4}
	3	2.59			{1; 2 < 5}
	4	2.92			{3 < 4}
	5	3.32			{3 < 5}
					{4 < 5}

### Multivariate Analyses

#### The Relationships between LLS and Foreign Language Attitude, LLS and Foreign Language Marks, and LLS and General School Achievement

Our results demonstrated that the sample was evaluated at an approximate level of mark 4 (*M*_Year5_ = 3.84, *SD*_Year5_ = 1.17; *M*_Year8_ = 3.62, *SD*_Year8_ = 1.17); however, Year 5 children achieved significantly higher (*p* < 0.01). As regards children’s attitudes, we found no significant differences between the years (*M*_Year5_ = 3.53, *SD*_Year5_ = 1.35; *M*_Year8_ = 3.43, *SD*_Year8_ = 1.23; *p* < 0.05). On the whole, it can be stated that children’s foreign language marks are higher than their attitude toward foreign language. The average school achievement showed significantly higher means than foreign language marks in both years (*M*_Year5_ = 3.82, *SD*_Year5_ = 0.87, *p* < 0.001; *M*_Year8_ = 3.62, *SD*_Year8_ = 1.17, *p* < 0.001).

We also examined the correlation between LLS and attitude toward foreign languages, LLS and the foreign language mark, and LLS and general school achievement. We observed the most significant estimates between language learning strategy use and attitude in Year 5 (*r* = 0.53–0.20; *p* < 0.001–0.05). The correlational coefficient between attitude and the foreign language mark was also significant (*r* = 0.37; *p* < 0.001). We noted that children who achieved higher in foreign languages showed a more positive attitude toward them. We also noticed a significantly strong effect for the foreign language mark and strategy use (*r* = 0.49–0.13; *p* < 0.001–0.05).

In Year 8, we found significant (*r*_Year5_ = 0.70–0.12; *p* < 0.001–0.01; *r*_Year8_ = 0.82–0.66; *p* < 0.001–0.01) relationships between overall strategy use and foreign language marks, attitudes and general school achievement. However, the relationship between affective strategies and school achievement was not significant. We observed that children who use LLS have positive attitudes toward language learning, except for compensation and affective strategies.

#### The Effect of Language Learning Strategies on Attitude, School Marks and General School Achievement

**FIGURE 1:**
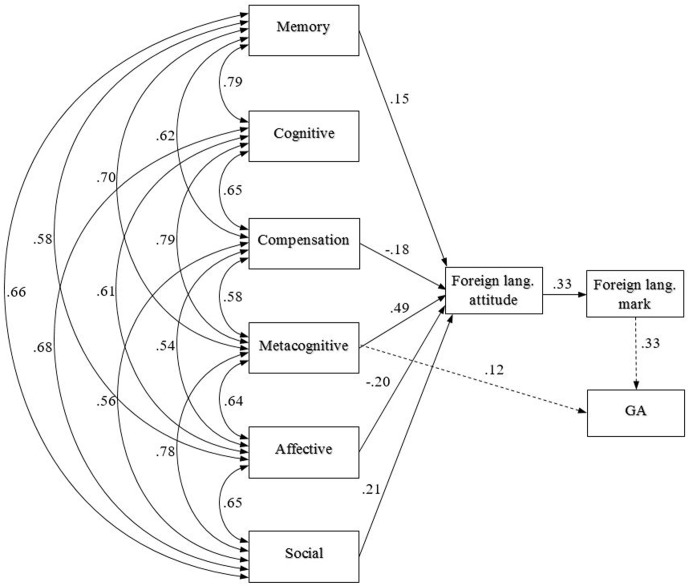
The path model for LLS influence on foreign language mark through foreign language attitude and general school achievement (GA) in Year 5.

**FIGURE 2:**
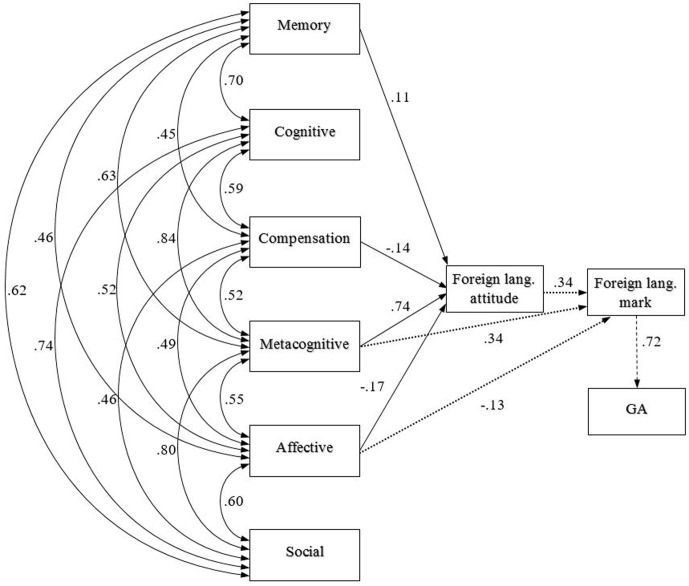
The path model for LLS influence on foreign language mark through foreign language attitude and general school achievement (GA) in Year 8.

We analysed the effect of LLS on foreign language attitude, school marks and general achievement using AMOS. We were looking for causalities between questionnaire fields and further variables by constructing a theoretical model on the basis of Oxford’s strategy taxonomy and children’s background data. We hypothesised that strategy factors largely influence children’s attitude toward language learning and through this the other variables. The model we created showed appropriate fit indices for the final model and indicated a good fit to our data in both years (**Figures 1**, **2**).

Year_5_: χ^2^(13) = 18,309, *p* = 0.146; Year_8_: χ^2^ (13) = 23,893, *p* = 0.18. An analysis of the hypothesised path model indicated a comparative fit index (CFI) of 0.998 in Year 5 and 0.994 in Year 8. The RMSEA (root mean squared error of approximation) was also good in both years, 0.030 in Year 5 and.049 in Year 8. Both the Tucker–Lewis index (TLI_Year5_ = 0.992; TLI_Year8_ = 0.981) and the normed fit index (NFI_Year8_ = 0.992; NFI_Year8_ = 0.989) confirmed that the model we constructed was a good fit to our data.

## Discussion

The main aim of the present study was to investigate our understanding of LLS in a foreign language learning context. Therefore, first, we identified the strategy use preferences in the sample and specified the most and least often used strategies among children with different proficiency levels. Second, we examined the children’s LLS use in connexion with their foreign language attitude, proficiency and general school achievement. Our results confirmed some results from previous studies and also established new relationships among the variables.

Regarding the general strategy use preferences of the sample, the students reported moderate use of the six strategy categories. The use of indirect strategies, more precisely, metacognitive, affective and social strategies, was the highest in Year 5, while metacognitive, cognitive and social strategies were the most frequently employed in Year 8. These findings shed light on the different preferences among the different ages and proficiency levels. While affective strategies play a significant role in Year 5, cognitive strategies become more dominant later. Metacognitive and social strategies remained the most frequently used in both Years. Our result is consistent with those reported by Dawadi (2017) who discovered similar strategy preferences. We can also reinforce Alhaysony’s (2017) results that high school sample did not engage in affective strategies, and Charoento’s (2016) findings about the low use of memory strategies.

We also examined the differences in strategy use among students with different proficiency levels in both Years. In Year 5 the research findings analysis demonstrated significant differences among strategy uses in four areas: the memory, cognitive, metacognitive and social fields. We noted no significant differences among children in compensation and affective strategies. As regards memory strategies, we observed that low-achieving children rarely employed them. Low achievers used cognitive strategies significantly less often than good and high performers. As our results showed, the most excellent learners are also metacognitive strategy users, and they engage in social strategies significantly very often. In Year 8, we observed significant differences in every field among children with different proficiencies. As in Year 5, the use of metacognitive and social strategies was the most frequent among the high-achieving students; however, cognitive strategy use was also relatively high. Charoento (2016) and Rao (2016) reported the same results, so we can confirm his previous research outcomes that high achievers avail themselves of strategies significantly more frequently than low-performing learners.

We also investigated the relationship between LLS and foreign language attitude, LLS and the foreign language mark, and LLS and general school achievement. According to our results, we found that children who prefer foreign language learning reported significantly higher strategy use. As regards foreign language marks, the relationships between different kinds of strategy users and their foreign language marks were low. Children with high proficiency did not necessarily employ each of the strategies at a higher rate. The same result was reached by Chen (2009). The relationship between affective strategies and school achievement was not significant. We observed that children who use LLS have positive attitudes toward language learning. So our findings partly confirmed previous results reported by Jabbari and Golkar (2014) and Platsidou and Kantaridou (2014).

Concerning the impact of strategy use on foreign language learning attitudes, proficiency and general school achievement. In Year 5 the effect of the questionnaire fields on foreign language attitude was considerably high; attitudes were strongly influenced by metacognitive strategies, and the effect of social strategies was also high. While memory and cognitive strategies showed positive paths to attitudes, compensation and affective strategies indicated negative effects on attitudes. Foreign language attitudes signified the same effect on foreign language marks as these marks did on general achievement. A lower but significant effect of metacognitive strategies was found on general school achievement in Year 5.

In Year 8, we found similar tendencies. The effect of metacognitive strategies on foreign language attitudes was very high, while that of memory strategies was low. The effect of social strategies was lost in Year 8. The impact of foreign language attitude on the foreign language mark was almost the same as in Year 5, but that of the foreign language mark on general school achievement was twice as high. Shawer (2016) likewise highlighted what our results have also shown: strategy use has a significant effect on general school achievement. Metacognitive strategies also had a direct effect on foreign language marks. On the whole, not only did we observe a strong use of metacognitive strategies, but the effect of metacognitive strategies on attitudes was also dominant in both years. Moreover, metacognitive strategies influenced school achievement in Year 5 and foreign language marks in Year 8.

To sum up, our results demonstrated that like other studies, our Hungarian sample showed significant preferences for metacognitive strategy use. Compensatory strategies were the least frequently preferred in Year 5 and memory strategies were the least common in Year 8, a finding which also reinforced previous research outcomes (Doró and Habók, 2013). We observed significant differences between more and less proficient students in strategy use. In line with other research (Platsidou and Kantaridou, 2014), we conclude that more proficient learners avail themselves of a broader range of strategies than less proficient students and strategy use has a significant effect on foreign language marks.

The research focused on the whole language process in connexion with several other factors among young students. The added value of our research is not only that we discovered relationships between factors required for foreign language learning, but direct and indirect underlying effects have also been brought to light through path analysis. These analyses provide a comprehensive view both of the dominant role of metacognitive strategies and of the foreign language learning process generally.

In spite of its value, the study has certain limitations. First, we employed a self-report instrument for data collection which does not address students’ deeper views on learning. Qualitative methods would make it possible to gain a more detailed understanding of foreign language learning through interviews, including think-aloud procedures and classroom observations. Second, the current research into LLS and proficiency among Hungarian students was conducted with participants from two different years at the lower secondary school level, so generalisation of the results is limited. In addition, our sample was not representative. Further research would be necessary to fully examine the relationship between language learning strategies, language learning attitudes, foreign language proficiency and general achievement among Hungarian students in a variety of years and in a larger sample.

Third, the current research only used two measurement points of proficiency, the foreign language mark and general achievement, which are evaluated by different teachers. In future, we will collect a wider range of language proficiency data, including language proficiency test and interviews. Fourth, a comparison of LLS and general learning strategies would produce a more nuanced overview of students’ strategy use.

## Conclusion and Pedagogical Implications

The main purpose of the present study was to ascertain the effect of LLS on other variables, such as foreign language attitude, foreign language proficiency and general school achievement among secondary school children in Hungary at the beginning and end of lower secondary school. In the beginner phase of learning foreign languages, it is important to better understand the relationship between language learning and related factors. Hence, our main objective was to provide a complex overview of these measurement points and to examine how LLS can support children in the first phase of the language learning process.

We used the Hungarian translation of Oxford’s Strategy Inventory for Language Learning questionnaire and supplemented it with the children’s self-reports of their foreign language attitudes and proficiency indicated by their foreign language mark and school achievement. This provided the basis for our research.

Past research has demonstrated that students with more frequent LLS use have better chances to become more proficient language learners. It has been pointed out that students that are more proficient engage in a wider range of strategies and select learning strategies dependent on learning tasks. Thus, teachers are encouraged to introduce a range of strategies for children to be able to select those that are most appropriate to features of their personality and relevant to learning tasks. At this age, introducing LLS is significant, particularly for children with low and average foreign language marks. It would be essential to motivate children to discover a variety of ways to practise their foreign language and find opportunities to read and engage in conversations with others. Children who are able to recognise the significance of language learning and use a broad range of strategies can find new ways and opportunities to practise language and to improve their proficiency. Hence, it would be highly recommended to integrate LLS consciously into foreign language lessons.

## Ethics Statement

This study was carried out in accordance with the recommendations of the University of Szeged. According to these recommendations participation in the study was voluntary both for schools and students. The participating schools had consent with the parents in allowing their students’ engagement in the research. According to the Hungarian law, the schools’ responsibility to conduct a written agreement with the parents about their consent to allow their children to take part in researches. The whole process is permitted and coordinated by the school holding municipalities. The agreements are documented and stored in written forms in the schools. The authors declare that data collection and handling strictly adhered to the usual standards of research ethics as approved by the University of Szeged.

## Author Contributions

AH and AM substantially contributed to the conception and design of the study, data collection, analysis and interpretation of data for the research. Both have written the manuscript and reviewed all parts of the manuscript. AH and AM have given final approval of the final version to be published. AH and AM agree to be accountable for all aspects of the work.

## Conflict of Interest Statement

The authors declare that the research was conducted in the absence of any commercial or financial relationships that could be construed as a potential conflict of interest.

## References

[B1] Alhaysony M. (2017). Language learning strategies use by Saudi EFL students: the effect of duration of English language study and gender. *Theory Pract. Lang. Stud.* 7 18–28. 10.17507/tpls.0701.03

[B2] Al-Qahtani M. F. (2013). Relationship between English language, learning strategies, attitudes, motivation, and students’ academic achievement. *Educ. Med. J.* 5 19–29. 10.5959/eimj.v5i3.124 12727095

[B3] Arbuckle J. L. (2008). *AMOS (Version 17.0) [Computer Software]*. Chicago, IL: SPSS.

[B4] Ardasheva Y. Tretter T. R. (2013). Strategy inventory for language learning–ELL student form: testing for factorial validity. *Mod. Lang. J.* 97 474–489. 10.1111/j.1540-4781.2013.12011.x

[B5] Byrne B. M. (2010). *Structural Equation Modelling Using AMOS. Basic Concepts, Applications, and Programming* 2nd Edn. New York: Routledge.

[B6] Chamot A. U. (2004). Issues in language learning strategy research and teaching. *Electron. J. Foreign Lang. Teachnol.* 1 14–26.

[B7] Charoento M. (2016). Individual learner differences and language learning strategies. *Contemp. Educ. Res. J.* 7 57–72.

[B8] Chen M. (2014). Age differences in the use of language learning strategies. *Engl. Lang. Teach.* 7 144–151. 10.5539/elt.v7n2p144

[B9] Chen M. L. (2009). Influence of grade level on perceptual learning style preferences and language learning strategies of Taiwanese English as a foreign language learners. *Learn. Individ. Dif.* 19 304–308. 10.1016/j.lindif.2009.02.004

[B10] Dawadi S. (2017). Language learning strategies profiles of EFL learners in Nepal. *Eur. J. Educ. Soc. Sci.* 2 42–55.

[B11] Doró K. Habók A. (2013). Language learning strategies in elementary school: the effect of age and gender in an EFL context. *J. Linguist. Lang. Teach.* 4 25–37.

[B12] Griffith C. Oxford R. (2014). The twenty-first century landscape of language learning strategies: introduction to this special issue. *System* 43 1–10. 10.1016/j.system.2013.12.009

[B13] Griffiths C. Incecay G. (2016). “New directions in language learning strategy research: engaging with the complexity of strategy use,” in *New Directions in Language Learning Psychology* eds Gkonou C. Tatzl D. Mercer S. (Berlin: Springer) 25–38. 10.1007/978-3-319-23491-5_3

[B14] Gunning P. Oxford R. L. (2014). Children’s learning strategy use and the effects of strategy instruction on success in learning ESL in Canada. *System* 43 82–100. 10.1016/j.system.2013.12.012

[B15] Jabbari M. J. Golkar N. (2014). The relationship between EFL learners’ language learning attitudes and language learning strategies. *Int. J. Linguist.* 6 161–167. 10.5296/ijl.v6i3.5837

[B16] Khaldieh S. A. (2000). Learning strategies and writing processes of proficient vs. less-proficient learners of Arabic. *Foreign Lang. Ann.* 33 522–533. 10.1111/j.1944-9720.2000.tb01996.x

[B17] Kline R. B. (2015). *Principles and Practice of Structural Equation Modeling* 4th Edn. New York, NY: Guilford Press.

[B18] Liu J. (2010). Language learning strategies and its training model. *Int. Educ. Stud.* 3 100–104. 10.5539/ies.v3n3p100

[B19] Magogwe J. M. Oliver R. (2007). The relationship between language learning strategies, proficiency, age, and self-efficacy beliefs: a study of language learners in Botswana. *System* 35 338–352. 10.1016/j.system.2007.01.003

[B20] Oxford R. L. (1990). *Language Learning Strategies: What Every Teacher Should Know*. Boston, MA: Heinle and Heinle.

[B21] Oxford R. L. (2016). *Teaching and Researching Language Learning Strategies: Self-Regulation in Context*. New York, NY: Routledge.

[B22] Oxford R. L. Burry-Stock J. A. (1995). Assessing the use of language learning strategies worldwide with the ESL/EFL version of the strategy inventory for language learning (SILL). *System* 23 1–23. 10.1016/0346-251X(94)00047-A

[B23] Pfenninger S. E. Singleton D. (2017). *Beyond Age Effects in Instructional L2 Learning: Revisiting the Age Factor*. Clevedon: Multilingual Matters. 10.21832/PFENNI7623

[B24] Platsidou M. Kantaridou Z. (2014). The role of attitudes and learning strategy use in predicting perceived competence in school-aged foreign language learners. *J. Lang. Lit.* 5 253–260. 10.7813/jll.2014/5-3/43

[B25] Platsidou M. Sipitanou A. (2014). Exploring relationships with grade level, gender and language proficiency in the foreign language learning strategy use of children and early adolescents. *Int. J. Res. Stud. Lang. Learn.* 4 83–96. 10.5861/ijrsll.2014.778

[B26] Rao Z. (2016). Language learning strategies and English proficiency: interpretations from information-processing theory. *Lang. Learn. J.* 44 90–106. 10.1080/09571736.2012.733886

[B27] Shang H. F. (2010). Reading strategy use, self-efficacy and EFL reading comprehension. *Asian EFL J.* 12 18–42.

[B28] Shawer S. F. (2016). Four language skills performance, academic achievement, and learning strategy use in preservice teacher training programs. *TESOL J.* 7 262–303. 10.1002/tesj.202

[B29] Wong L. L. C. Nunan D. (2011). The learning styles and strategies of effective language learners. *System* 39 144–163. 10.1016/j.system.2011.05.004 19289721

[B30] Wu Y. L. (2008). Language learning strategies used by students at different proficiency levels. *Asian EFL J.* 10 75–95. 17711614

